# Decreased choroidal and scleral thicknesses in highly myopic eyes with posterior staphyloma

**DOI:** 10.1038/s41598-021-87065-6

**Published:** 2021-04-12

**Authors:** Un Chul Park, Eun Kyoung Lee, Bo Hee Kim, Baek-Lok Oh

**Affiliations:** 1 Department of Ophthalmology, Seoul National University College of Medicine, 103 Daehak-ro, Jongno-gu, Seoul, 110-799 Korea; 2 Retinal Degeneration Research Laboratory, Seoul National University Hospital Biomedical Research Institute, Seoul, Korea

**Keywords:** Anatomy, Medical research

## Abstract

In this cross-sectional study, we investigated choroidal thickness (CT) and scleral thickness (ST) in highly myopic eyes and their associations with ocular factors. Patients underwent widefield swept-source optical coherence tomography (OCT) to measure the CT and ST at the subfovea and 3000 μm superior, inferior, temporal, and nasal to the fovea and macular curvature. A total of 237 eyes (154 patients) were included. At all five measurement points, thinner CTs and STs were associated with longer axial lengths (*r* = − 0.548 to − 0.357, all *P* < 0.001) and greater macular curvatures (*r* = − 0.542 to − 0.305, all *P* < 0.001). The CT and ST were significantly thinner in eyes with posterior staphyloma than in those without at all measurement points (all *P* ≤ 0.006) but did not differ between eyes with the wide macular and narrow macular type of staphyloma. Eyes with myopic maculopathy of category ≥ 3 according to the International Meta-Analysis for Pathologic Myopia classification had significantly thinner CTs and STs than those with category ≤ 2 (all *P* ≤ 0.005). In highly myopic eyes, a decrease in the CT and ST was more pronounced in eyes with more structural changes, such as longer axial length, steeper macular curvature, and the presence of posterior staphyloma.

## Introduction

Pathologic myopia is one of the leading causes of irreversible visual impairment worldwide, particularly in East Asian countries where myopia is highly prevalent^1–3^. Visual impairment is mainly caused by myopic maculopathy, which refers to a range of chorioretinal changes in the posterior pole. Myopic maculopathy has been shown to progress over time to a higher category of atrophic change, leading to significant visual loss^4–6^. Although several clinical and ocular factors have revealed to have a significant association with the progression of myopic maculopathy, little is known about the exact mechanism involved in the development and progression of myopic maculopathy.

Recent observational longitudinal studies have shown that a greater axial elongation is significantly associated with the progression of myopic maculopathy, which suggests that structural change in the eyeball may be a major contributing factor for myopic maculopathy^4,7^. In addition, posterior staphyloma, which indicates the localized outpouching of the eyeball wall at the posterior pole with a radius of less than the surrounding curvature, is associated with a higher prevalence of macular complications such as chorioretinal atrophy, myopic choroidal neovascularization, and myopic traction maculopathy^8,9^.

In this context, structural evaluation of the posterior pole may be helpful in elucidating the pathogenesis of macular complications in pathologic myopia. Furthermore, the influence of posterior staphyloma on choroidal thickness (CT) or scleral thickness (ST) remains unknown. In this study, we evaluated the association of CT and ST with the structural changes related to high myopia, especially the presence of posterior staphyloma.

## Methods

### Study population

In this cross-sectional study, the medical records of patients with high myopia who were consecutively examined using swept-source optical coherence tomography (OCT) at the Retina Center of the Seoul National University Hospital between January 2018 and July 2020 were reviewed. High myopia was defined as an axial length of ≥ 26.5 mm or a myopic refractive error ≥ − 6.0 diopters. The exclusion criteria were as follows: (1) poor quality of the swept-source OCT image, that is, image not clear due to eye movement or media opacity and image that did not include subfoveal choroid and sclera within the scan depth or not centered on the fovea; (2) other retinal disorders, such as diabetic retinopathy, retinal vascular diseases, age-related macular degeneration, and central serous chorioretinopathy; (3) a previous history of vitreoretinal surgery, photodynamic therapy, and anti-vascular endothelial growth factor (VEGF) treatment; or (4) active myopic choroidal neovascularization. The protocol and study design were approved by the Institutional Review Board of the Seoul National University Hospital (IRB no. 2003-231-1115), and this study was conducted in accordance with the tenets of the Declaration of Helsinki. Informed consent was obtained from each participant for the study protocol and publication of identifying images in an online open-access publication.

### Clinical examination

All patients underwent a comprehensive ophthalmic examination including best-corrected visual acuity (BCVA), slit lamp examination, fundus examination, refractive error (spherical equivalent), axial length measurement, ultra-widefield retinal imaging, and widefield swept-source OCT. The axial length measurement was performed with the ocular biometry (IOLMaster; Carl Zeiss Meditec, Jena, Germany), and the ultra-widefield retinal image was taken with an Optos 200Tx scanning laser ophthalmoscope (Optos PLC, Scotland, UK) centered on the macula. Based on ultra-widefield retinal images, posterior staphyloma was determined by the presence of abnormal pigmentary changes in the pseudo-color image or an abnormal reflex in the red or green separation image indicating the staphyloma border^8,10^. When the presence of posterior staphyloma was ambiguous, widefield swept-source OCT images were also assessed to detect the staphyloma border, which is characterized by a gradual thinning of the choroid and an inward protrusion of the sclera^11^. The type of posterior staphyloma was determined based on the classification by Ohno-Matsui^8^, which classified the posterior staphyloma into five types (wide macular, narrow macular, peripapillary, nasal, and inferior) and others, according to its location and extent. According to the Meta-analysis of Pathologic Myopia (META-PM) Study Group classification, myopic maculopathy was classified into four categories: category 1, tessellated fundus; category 2, diffuse chorioretinal atrophy; category 3, patchy chorioretinal atrophy; and category 4, macular atrophy^12^. Three additional features, including lacquer cracks, Fuchs spots, and myopic choroidal neovascularization, were defined as plus lesions to supplement these categories.

### Swept-source OCT measurement

Widefield swept-source OCT examination was performed using the PLEX Elite 9000 (Carl Zeiss Meditec, Dublin, CA) with an HD spotlight B-scan with a 16-mm scan length and a 3-mm scan depth. Some patients were examined using a protocol with a 6-mm scan depth after the OCT machine upgrade. Our scanning protocol included a pattern of radial scans along six meridians centered on the fovea. A single image was made up of 1024 A-line scans and averaged 100 times to yield a high-quality image. The thickness of the sclera and choroid was measured manually using a caliper provided by the OCT machine at the subfoveal region and 3000 μm nasal, temporal, superior, and inferior to the fovea center. Considering that the dimension in the OCT images are affected by long axial length, measurement points of each eye were determined based on a previously reported formula: *t* = 3.382 × 0.01306 × (axial length – 1.82) × *s*, where *t* and *s* represent the actual dimensions and measurements in the OCT image, respectively^13^. The CT was defined as the perpendicular distance between the outer edge of the hyperreflective line of the retinal pigment epithelium (RPE) and the choroid-scleral junction. The OCT images were maximally magnified when the choroid was extremely thin, but CT was determined as 0 μm at points where the choroid was not visible. The ST was defined as the perpendicular distance between the choroid-scleral junction and outer scleral border. The thickness of the episclera, which appeared as a relatively uniform structure with a slightly lower reflectivity than the sclera, was not included in the ST measurement^14^. When a definite linear hyporeflective structure running within the sclera along its curvature, which suggests short or long posterior ciliary artery^15^, was observed, the hyporeflective portion within the sclera was not included in the ST. In addition, the curvature index, which is a recently proposed parameter to assess the macular curvature in myopic eyes^16^, was measured using the ImageJ software (NIH, Bethesda, MD). As a ratio of RPE line length compared to the straight distance between two points 6 mm apart centered on the fovea, greater curvature index represented a steeper macular curvature with more protrusion (see Supplementary Figure S1 online). The curvature indices obtained using six radial B scan images were averaged for analysis. In the analysis of subfoveal ST and curvature index, eyes with dome-shaped macula were excluded, which was defined as an inward bulge of the macular RPE of > 50 μm above a presumed line of the RPE line at the bottom of the macular curvature.

### Statistical analysis

Two independent retinal specialists (BO and BHK) performed the grading of myopic maculopathy and posterior staphyloma and the measurement of CT, ST, and curvature index in a masked manner. Any discrepancies were adjudicated by a senior retinal specialist (UCP), and the measurements from the two graders were averaged for statistical analysis. Interobserver reproducibility of the CT, ST, and curvature index was assessed using intraclass correlation coefficients. Considering the invisibility of the posterior scleral border in some eyes, clinical and ocular characteristics were compared between eyes with and without any immeasurable points of ST. Correlations of CT and ST with age, axial length, and curvature index were evaluated. The CT, ST, and curvature index were compared according to the categories of myopic maculopathy or the presence of a posterior staphyloma using one-way analysis of variance or *t*-test, respectively. Post hoc analysis was performed using the Bonferroni method. All statistical analyses were performed using SPSS software version 22.0 (SPSS Inc., Chicago, IL), and *P* values of < 0.05 were considered statistically significant.

## Results

Of the 372 eyes of 189 highly myopic patients who underwent swept-source OCT examination during the study period, 135 eyes were excluded: 40 eyes due to the poor quality of OCT images, 11 due to the presence of other chorioretinal disorders, 41 due to a history of intravitreal anti-VEGF treatment or photodynamic therapy, and 43 due to a history of vitreoretinal surgery. In total, 237 eyes of 154 patients were included in the analysis. The mean age was 63.0 ± 11.6 years (range, 24–89), and the mean axial length was 30.4 ± 2.3 mm (range, 25.1–37.1). Baseline demographic and ocular characteristics of the study eyes are listed in Table 1. According to the META-PM Study Group classification, 63 (26.6%), 95 (40.1%), 61 (25.7%), and 18 (7.6%) eyes were graded as having myopic maculopathy of category 1 (fundus tessellation), category 2 (diffuse chorioretinal atrophy), category 3 (patchy chorioretinal atrophy), and category 4 (macular atrophy), respectively. Patients with a higher category of myopic maculopathy tended to be older, have a longer axial length, a greater myopic refractive error, and worse visual acuity. A plus lesion was present in 74 eyes (31.2%). On swept-source OCT images, a dome-shaped macula was observed in 39 eyes (16.5%).

**Table 1:** Baseline characteristics and demographics of patients.

Parameters	Total	Category 1 (fundus tessellation)	Category 2 (diffuse atrophy)	Category 3 (patchy atrophy)	Category 4 (macular atrophy)	*P*-value
Number of eyes (%)/patients	237 eyes (100.0%)/154 patients	63 eyes (26.6%)	95 eyes (40.1%)	61 eyes (25.7%)	18 eyes (7.6%)	–
Mean age, years (range)	63.0 ± 11.6 (24 to 89)	59.4 ± 14.3 (24 to 89)	61.9 ± 10.6 (38 to 85)	64.8 ± 9.9 (38 to 81)	69.5 ± 7.7 (34 to 83)	0.003
Gender, number of eyes (%) of women	198 eyes (83.5%)/128 women	47 eyes (74.6%)	80 eyes (84.2%)	54 eyes (88.5%)	17 eyes (94.4%)	0.092
Mean axial length, mm (range)	30.4 ± 2.3 (25.1 to 37.1)	28.4 ± 1.6 (25.1 to 31.4)	30.4 ± 2.0 (25.7 to 34.9)	31.9 ± 2.0 (28.4 to 37.1)	31.1 ± 2.4 (28.2 to 35.3)	< 0.001
Mean refractive error, diopter (range)^a^	− 15.4 ± 5.4 (− 5.5 to − 25.0)	− 11.1 ± 5.5 (− 5.5 to − 25.0)	− 15.7 ± 4.6 (− 7.1 to − 23.8)	− 16.2 ± 5.2 (− 9.3 to − 23.5)	− 14.7 ± 4.3 (− 8.5 to − 20.3)	0.003
Mean baseline BCVA, logMAR (range, Snellen equivalent)	0.55 ± 0.55 (− 0.18 to 2.50, 20/71)	0.30 ± 0.28 (− 0.18 to 1.00, 20/40)	0.46 ± 0.40 (− 0.08 to 2.00, 20/58)	0.60 ± 0.56 (− 0.08 to 2.50, 20/80)	1.66 ± 0.64 (0.30 to 2.50, 20/914)	< 0.001
Myopic choroidal neovascularization	18 (7.6%)	1 (1.6%)	3 (3.2%)	3 (4.9%)	11 (61.1%)	< 0.001
Lacquer crack	58 (24.5%)	13 (20.6%)	29 (30.5%)	16 (26.2%)	0 (0.0%)	0.038
Dome-shaped macula	39 (16.5%)	0 (0.0%)	15 (15.8%)	21 (34.4%)	3 (16.7%)	< 0.001
Presence of posterior staphyloma	169 (71.3%)	30 (47.6%)	71 (74.7%)	53 (86.9%)	15 (83.3%)	< 0.001

*BCVA* best-corrected visual acuity, *logMAR* logarithm of minimal angle of resolution.

^a^Phakic eyes without history of refractive surgery.

Of the 1185 thickness measurement points in the 237 eyes, the entire thickness of the choroid was visible at all points. However, the posterior border of the sclera could not be detected on swept-source OCT images in 162 (13.7%) points in 73 eyes (30.8%); thus, ST was not measurable. These points were most frequently observed in eyes with myopic maculopathy of category 1 (113 points, 69.8%), followed by those with category 2 (40 points, 24.7%), 3 (6 points, 3.7%), and 4 (3 points, 1.9%). Topographically, ST was not measurable at the subfoveal, superior, inferior, temporal, and nasal regions in 21 (8.9%), 54 (22.8%), 20 (8.4%), 47 (19.8%), and 20 (8.4%) eyes, respectively. When compared to the 164 eyes whose ST was measurable at all points, 73 eyes with one or more immeasurable points had a significantly shorter axial length (*P* < 0.001), lower category of myopic maculopathy (*P* < 0.001), lower frequency of posterior staphyloma (*P* = 0.028), and lower curvature index (*P* = 0.004). When comparing measurable thicknesses, eyes with any point of immeasurable ST had a significantly greater CT and ST at all points except the nasal CT (Table 2).

**Table 2:** Comparison of clinical characteristics according to the visibility of posterior scleral border.

Parameters	Visibility of posterior scleral border		*P*-value
	Visible at all points	Invisible at any points	
Number of eyes (%)	164 (69.2%)	73 (30.8%)	–
Mean age, years (range)	62.4 ± 11.0 (24–84)	63.0 ± 12.9 (27–89)	0.739
Mean axial length, mm (range)	31.1 ± 2.1 (25.8–37.1)	28.8 ± 2.0 (25.1–33.8)	< 0.001
Mean baseline BCVA, logMAR (range, Snellen equivalent)	0.62 ± 0.58 (− 0.08 to 2.50, 20/83)	0.38 ± 0.44 (− 0.18 to 2.50, 20/48)	0.003
**Category of myopic maculopathy**			
Category 1 (fundus tessellation)	22 (13.4%)	41 (56.2%)	< 0.001
Category 2 (diffuse atrophy)	71 (43.3%)	24 (32.9%)	
Category 3 (patchy atrophy)	55 (33.5%)	6 (8.2%)	
Category 4 (macular atrophy)	16 (9.8%)	2 (2.7%)	
**Choroidal thickness, μm (range)**			
Subfoveal	25.0 ± 16.2 (0–95)	55.1 ± 39.0 (0–187)	< 0.001
Superior	47.1 ± 32.1 (0–178)	90.9 ± 52.7 (0–245)	< 0.001
Inferior	37.8 ± 28.2 (0–153)	71.3 ± 39.2 (0–190)	0.008
Temporal	41.9 ± 34.3 (0–165)	82.5 ± 52.0 (0–257)	< 0.001
Nasal	23.9 ± 19.1 (0–137)	40.3 ± 24.5 (0–149)	0.083
**Scleral thickness, μm (range)**^**a**^			
Subfoveal^b^	261.2 ± 86.9 (103–501)	336.8 ± 87.1 (125–570)	< 0.001
Superior	237.6 ± 73.5 (90–517)	361.1 ± 76.8 (210–522)	< 0.001
Inferior	227.0 ± 61.5 (93–447)	290.9 ± 77.1 (118–469)	< 0.001
Temporal	229.4 ± 71.8 (70–448)	297.1 ± 89.1 (153–469)	< 0.001
Nasal	257.2 ± 78.2 (69–488)	291.8 ± 64.8 (120–492)	0.003
Presence of posterior staphyloma	124 (75.6%)	45 (61.6%)	0.028
Curvature index (range)^b^	1.049 ± 0.032 (1.011–1.241)	1.035 ± 0.027 (1.007–1.181)	0.003

*BCVA* best-corrected visual acuity, *logMAR* logarithm of minimal angle of resolution.

^a^Included only points where scleral thickness was measurable.

^b^Excluded eyes with dome-shaped macula.

The CT and ST at all measurement points showed significant correlations with axial length and curvature index (all *P* < 0.001; Table 3); a thin choroid and sclera were associated with a longer axial length and greater curvature index. Age was significantly correlated with subfoveal, superior, and temporal CT, but not with ST at any point. Significant correlations between CT and ST were found at all five measurement points (all *P* < 0.01). At five measurement points, interobserver intraclass correlation coefficients were ≥ 0.892 for CTs and ≥ 0.901 for measurable STs. Interobserver intraclass correlation coefficients for the curvature index were 0.920 (see Supplementary Table S1 online).

**Table 3:** Correlation coefficients for the associations of choroidal and scleral thicknesses with age, axial length, and curvature index.

Parameters		Age	Axial length	Curvature Index^a^	Scleral thickness**
Choroidal thickness	Subfoveal	− 0.224^†^	− 0.429^‡^	− 0.351^‡^	0.278^a,^^†^
	Superior	− 0.260^‡^	− 0.479^‡^	− 0.362^‡^	0.389^‡^
	Inferior	− 0.108	− 0.548^‡^	− 0.321^‡^	0.433^‡^
	Temporal	− 0.227^‡^	− 0.541^‡^	− 0.378^‡^	0.403^‡^
	Nasal	− 0.035	− 0.537^‡^	− 0.305^‡^	0.179^†^
Scleral thickness	Subfoveal^a^	0.053	− 0.424^‡^	− 0.542^‡^	–
	Superior	− 0.051	− 0.428^‡^	− 0.423^‡^	–
	Inferior	− 0.034	− 0.505^‡^	− 0.423^‡^	–
	Temporal	0.042	− 0.543^‡^	− 0.339^‡^	–
	Nasal	− 0.016	− 0.357^‡^	− 0.408^‡^	–
Curvature index^a^		0.019	0.458^‡^	–	–

^†^*P*-value of correlation coefficient < 0.01.

^‡^*P*-value of correlation coefficient < 0.001.

^a^Excluded eyes with dome-shaped macula.

Based on ultra-widefield retinal images, posterior staphyloma was observed in 169 eyes (71.3%). The wide macular type was most commonly observed in 102 eyes (60.4%), followed by narrow macular type in 53 eyes (31.4%), peripapillary type in 7 eyes (4.1%), inferior type in 5 eyes (3.0%), and nasal type in 2 eyes (1.2%). Eyes with posterior staphyloma had a significantly longer axial length (*P* < 0.001), a lower CT (*P* ≤ 0.003) and ST (*P* ≤ 0.006) at all measurement points, and a greater curvature index (*P* < 0.001) compared to those without posterior staphyloma (Table 4). However, there were no significant differences in axial length, CT, ST, and curvature index between the eyes with wide macular and narrow macular type posterior staphyloma (See Supplementary Table S2 online). In eyes with wide macular type staphyloma, four eyes showed combined staphylomas of peripapillary and narrow macular type. At all five measurement points, the CTs and STs of these four eyes were below the average of wide macular type staphyloma.

**Table 4:** Choroidal and scleral thicknesses according to the presence of posterior staphyloma.

Parameters	Posterior staphyloma		*P*-value
	Absent	Present	
Number of eyes (%)	68 (28.7%)	169 (71.3%)	–
Mean age, years (range)	62.0 ± 13.9 (27–89)	62.8 ± 10.6 (24–85)	0.616
Gender, number of eyes (%) of women	55 (80.9%)	143 (84.6%)	0.483
Mean axial length, mm (range)	29.3 ± 1.5 (25.9–32.4)	30.8 ± 2.4 (25.1–37.1)	< 0.001
Mean baseline BCVA, logMAR (range, Snellen equivalent)	0.47 ± 0.54 (− 0.18 to 2.50, 20/59)	0.58 ± 0.56 (− 0.18 to 2.50, 20/76)	0.206
Dome-shaped macula	7 (10.3%)	32 (18.9%)	0.105
**Choroidal thickness, μm (range)**			
Subfoveal	46.9 ± 39.3 (0–187)	29.2 ± 21.7 (0–133)	< 0.001
Superior	92.4 ± 51.7 (0–245)	47.8 ± 34.0 (0–172)	< 0.001
Inferior	68.3 ± 37.2 (0–190)	40.0 ± 31.5 (0–163)	< 0.001
Temporal	82.2 ± 47.5 (0–257)	43.2 ± 38.8 (0–215)	< 0.001
Nasal	38.7 ± 22.7 (0–149)	25.2 ± 21.5 (0–141)	0.003
**Scleral thickness, μm (range)**			
Subfoveal^a^	316.2 ± 76.5 (151–570)	268.7 ± 95.9 (103–542)	0.002
Superior	304.0 ± 81.5 (157–522)	230.3 ± 76.5 (90–460)	< 0.001
Inferior	276.7 ± 78.9 (93–455)	231.0 ± 67.0 (101–469)	< 0.001
Temporal	276.3 ± 65.9 (140–411)	226.6 ± 77.7 (70–469)	< 0.001
Nasal	288.9 ± 69.1 (139–488)	257.1 ± 76.9 (69–492)	0.006
Curvature index (range)^a^	1.030 ± 0.015 (1.007–1.072)	1.051 ± 0.033 (1.008–1.241)	< 0.001

*BCVA* best-corrected visual acuity, *logMAR* logarithm of minimal angle of resolution.

^a^Excluded eyes with dome-shaped macula.

**Figure 1:**
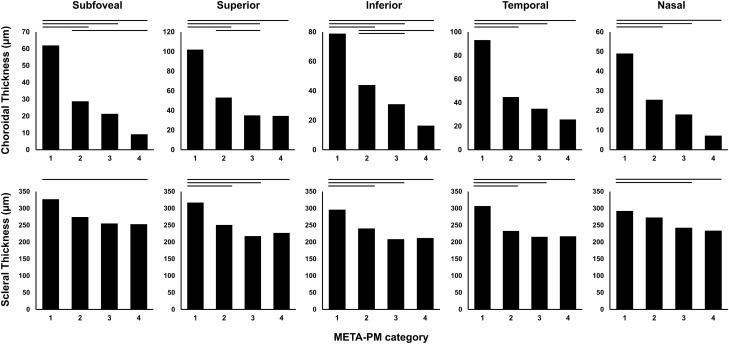
Choroidal and scleral thicknesses at the subfovea and at 3000 μm superior, inferior, temporal, and nasal to the fovea according to the category of myopic maculopathy based on the Meta-analysis of Pathologic Myopia Study Group classification. Horizontal bars indicate statistically significant differences between the categories after correction for multiple comparisons.

**Figure 2:**
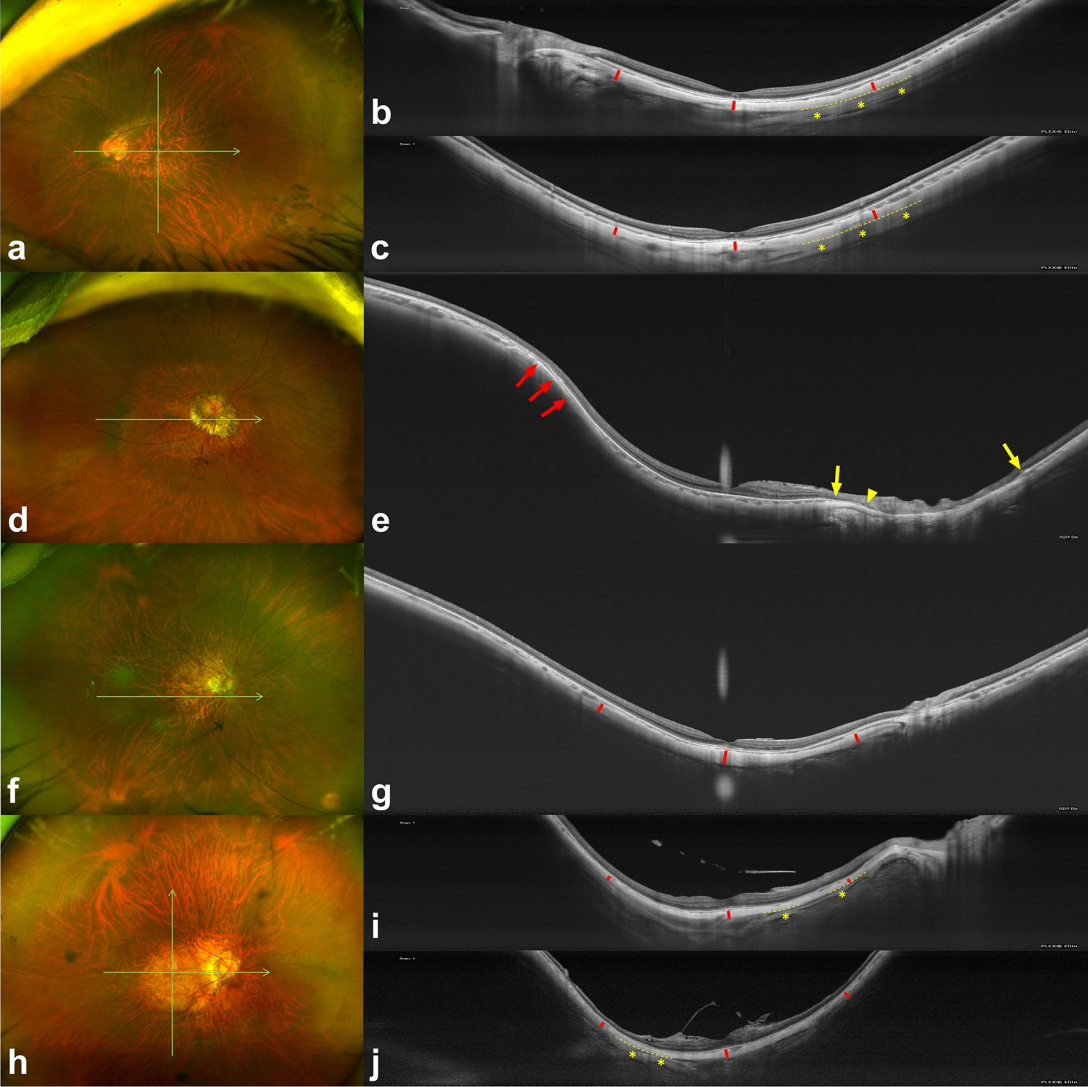
Images of highly myopic eyes with myopic maculopathy of category 1 (tessellated fundus) or 2 (diffuse chorioretinal atrophy). The green arrows represent the cross-section through which optical coherence tomography (OCT) scan was performed, and red bars represent scleral thicknesses at the fovea and at 3 mm from the fovea. In some images, the episcleral tissue appears as a uniform structure outside of the sclera with a slightly lower reflectivity (asterisks). Interfaces between the sclera and episcleral tissue are outlined with dashed lines. All OCT images were shown on a 1:1 µm scale. (**a**–**c**) A 24-year-old woman with tessellated fundus. The axial length was 30.63 mm and the best-corrected visual acuity (BCVA) was 20/32. The choroidal and scleral thicknesses at the foveal, superior, inferior, temporal, and nasal regions were 13, 122, 71, 81, 49 µm and 214, 234, 193, 205, 249 µm, respectively. (**d**,**e**) A 78-year-old woman with a mild peripapillary diffuse chorioretinal atrophy temporal to the disc. Axial length was 29.86 mm and BCVA was 20/25. Choroidal thickness at the foveal, temporal, and nasal regions was 55, 92, and 42 µm, respectively, but the posterior scleral border was not visible at any measurement point. An inward protrusion of the sclera and thinning of the choroid are observed at the edge of the staphyloma (red arrows). Note the margin of retinal pigment epithelium defects (yellow arrows) and end of the Bruch membrane (arrowhead). (**f**,**g**) A 63-year-old woman with peripapillary diffuse chorioretinal atrophy. Axial length was 30.07 mm and BCVA was 20/20. Choroidal and scleral thicknesses at the foveal, temporal, and nasal regions were 20, 51, 17 µm and 290, 211, 203 µm, respectively. (**h–j**) A 49-year-old woman with macular diffuse chorioretinal atrophy. Axial length was 34.55 mm and BCVA was 20/125. The choroidal and scleral thicknesses at the foveal, superior, inferior, temporal, and nasal regions were 19, 11, 12, 18, 14 µm and 197, 168, 143, 115, 104 µm, respectively.

**Figure 3:**
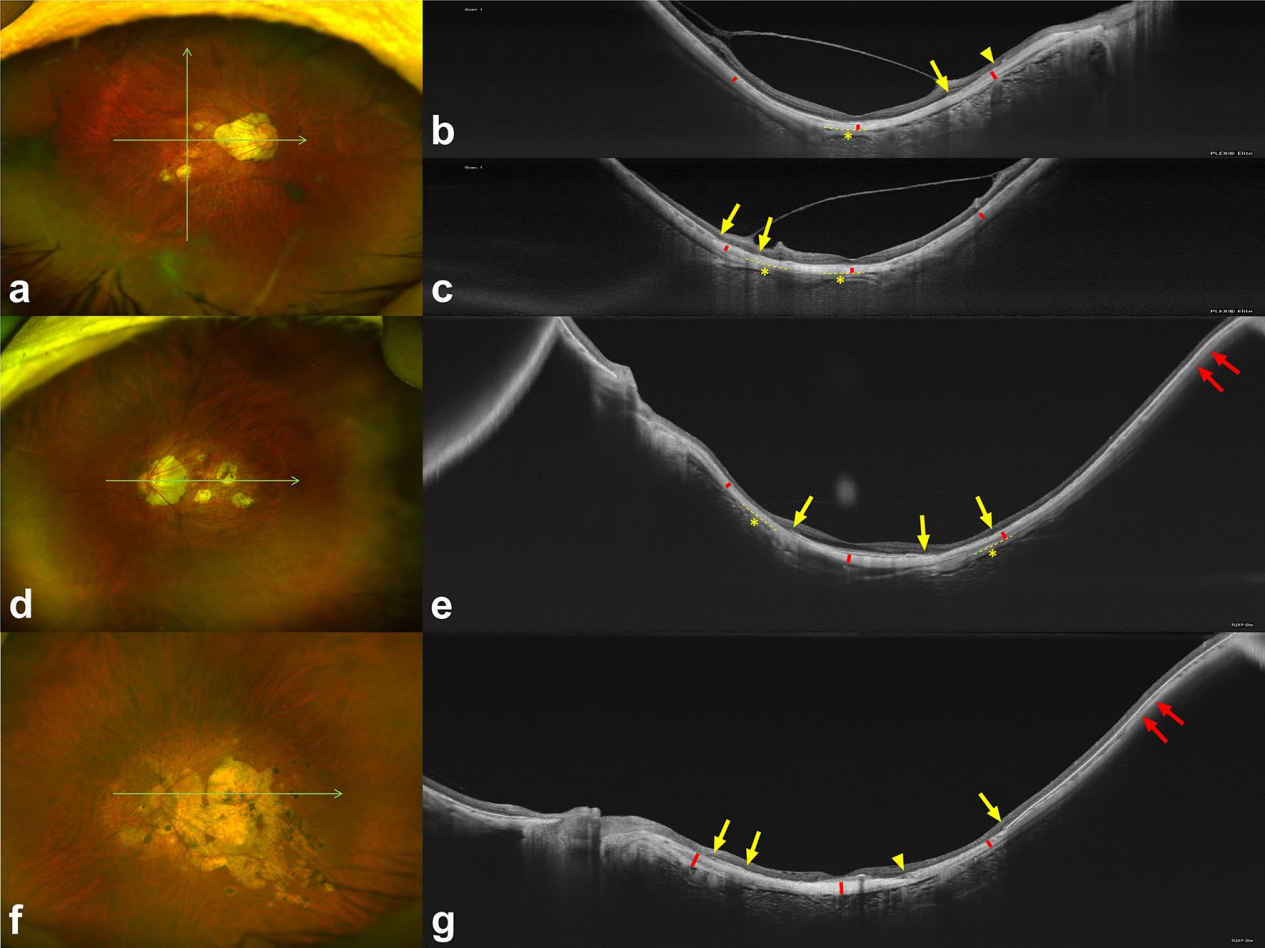
Images of highly myopic eyes with myopic maculopathy of category 3 (patchy chorioretinal atrophy) or 4 (macular atrophy). The green arrows represent the cross-section through which optical coherence tomography (OCT) scan was performed, and red bars represent scleral thicknesses at the fovea and 3 mm from the fovea. In some images, the episcleral tissue appears as a uniform structure outside of the sclera with a slightly lower reflectivity (asterisks). Interfaces between the sclera and episcleral tissue are outlined with dashed lines. All OCT images were shown on a 1:1 µm scale. (**a**–**c**) A 74-year-old man with patchy chorioretinal atrophy. The axial length was 33.92 mm and best-corrected visual acuity (BCVA) was 20/63. The choroidal and scleral thicknesses at the foveal, superior, inferior, temporal, and nasal regions were 15, 25, 34, 21, 0 µm and 108, 126, 101, 104, 168 µm, respectively. Note the margin of retinal pigment epithelium defects (arrows) and end of Bruch membrane (arrowhead). (**d**,**e**) A 71-year-old woman with patchy chorioretinal atrophy. Axial length was 32.42 mm and BCVA was 20/40. Choroidal and scleral thicknesses at the foveal, temporal, and nasal regions were 30, 15, 0 µm and 160, 156, 126 µm, respectively. Gradual thinning of the choroid is observed at the edge of the staphyloma (red arrows). Note the margin of retinal pigment epithelium defects (yellow arrows). (**f**,**g**) A 59-year-old man with choroidal neovascularization-related macular maculopathy. Axial length was 29.53 mm and visual acuity was count finger. On OCT, subretinal hyperreflective lesions at the fovea and extensive loss of the choroid and retinal pigment epithelium-Bruch membrane complex within an area of macular atrophy are observed. Note the margin of retinal pigment epithelium defects (yellow arrows) and crumpled remnants of the Bruch membrane (arrowhead). Gradual thinning of the choroid is observed at the edge of the staphyloma (red arrows). Choroidal and scleral thicknesses at the foveal, temporal, and nasal regions were 0, 0, 0 µm and 217, 145, 226 µm, respectively.

Figure 1 shows the CT and ST at five measurement points according to the categories of myopic maculopathy. For all parameters, one-way analysis of variance showed significant differences among the four categories. Eyes with category 1 had the greatest CT and ST at all points, while those with higher categories had lower thicknesses. Post hoc analysis by the Bonferroni method showed that category 1 differed from the other categories in all thickness parameters, except for category 2 in the subfoveal ST and nasal ST and from category 3 in subfoveal ST. Among the other categories, some parameters were different between categories 2 and 3 or between categories 2 and 4, but not between categories 3 and 4. In 63 eyes with myopic maculopathy of META-PM category 1 in this study, 50 eyes (79.4%) satisfied the cut-off CT of either 62 μm at the subfovea to diagnose macular diffuse choroidal atrophy or 56.5 μm nasal to the fovea to diagnose peripapillary diffuse choroidal atrophy, as suggested in a recent OCT-based study^17^. In consideration of this finding, eyes with myopic maculopathy of category ≤ 2 and ≥ 3 were compared, and those with category ≤ 2 had significantly greater CTs and STs at all measurement points (all *P* ≤ 0.005) and lower curvature index (*P* = 0.003). Representative OCT images of each category of myopic maculopathy are presented in the Figs. 2 and 3.

## Discussion

In this study, swept-source OCT imaging of highly myopic eyes revealed that decreased CT and ST were associated with a longer axial length and steeper macular curvature. In addition, CT and ST at all measurement points were significantly lower in eyes with posterior staphyloma than in those without. These findings suggest that structural alterations related to high myopia are associated with the decreased thickness of the choroid and sclera.

Swept-source OCT uses a longer wavelength light source, and improved tissue penetration allows for high-contrast imaging of the sclera and retrobulbar structures^18^. Compared to enhanced depth imaging by spectral-domain OCT, swept-source OCT allows better visualization of the entire thickness of the sclera, which is thought to be limited by the thickness of the choroid^19,20^. In addition, it has a longer scan width and depth compared to conventional OCT. With the use of widefield swept-source OCT in this study, the presence of posterior staphyloma could be determined more reliably. More importantly, its longer scan depth enabled the measurements of CT and ST at the regions 3000 μm from the fovea, even in eyes with a severely protruded posterior pole, which are usually out of the scan window and inverted within the image with conventional OCT. In this study, the entire thickness of the sclera was not visible in 13.7% of the measurement points, and 30.8% of patients had one or more points with immeasurable ST. This was comparable to the results of previous studies using swept-source OCT, which reported that the posterior scleral border was visible in 57–85% of highly myopic eyes but was less frequently or not visible in eyes with mild myopia or emmetropia^19–21^.

In a swept-source OCT study by Wong et al.^21^, the choroid and sclera were thinner in eyes with myopic maculopathy of category ≥ 3 than in eyes with category ≤ 2 at multiple points in the posterior pole. However, the statistical significance was observed only in the subfoveal CT. In contrast, our results showed significantly greater CTs and STs at all measurement points, and this discrepancy may be due to the larger sample size in the present study. Based on the stronger correlation of CT with the severity of myopic maculopathy compared to ST, the authors considered vascular factors to be of more importance in the pathogenesis of myopic degeneration^21^. However, the results of the present study suggest that mechanical or structural changes reflected by scleral thinning also play an important role. The mechanism of choroidal and scleral thinning in axially elongated eyes has not been fully elucidated. The sclera has long been considered as the primary tissue of axial elongation based on the histologic and molecular findings of reduced diameter of collagen fibrils and decreased collagen synthesis in the sclera in animal models of myopia^22–25^. Recently, Jonas et al. suggested a plausible novel theory that axial elongation in highly myopic eyes may result from the production of Bruch membrane in the equatorial region and subsequent backward pushing of the Bruch membrane, which leads to the compression of the choroid and secondary thinning of the sclera^26^. Even in this hypothesis, the biomechanical property of the sclera seems to play an important role in the enlargement of the eyeball posterior to the equator, particularly the development of posterior staphyloma. A recent histological study on enucleated human eyes suggested that there is a possibility that local outpouching of the sclera may result from locally reduced scleral resistance caused by the altered arrangement of collagen fibril bundles^27^.

In this study, the thicknesses of the choroid and sclera at all measurement points were negatively correlated with the axial length and steepness of the posterior pole, which was represented by the curvature index. In addition, the present study shows that the choroid and sclera in eyes with posterior staphyloma were significantly thinner at all measurement points than in eyes without, confirming the histological findings that revealed decreased ST at the center of staphyloma in the enucleated highly myopic eyes^27^. To our knowledge, this is the first study that measured ST in vivo in highly myopic eyes with posterior staphyloma compared to those without, using swept-source OCT. These results suggest that highly myopic eyes with more protrusion at the posterior pole, particularly in the presence of localized outpouching of the posterior staphyloma, have a thinner sclera and choroid. Thinner choroid in eyes with posterior staphyloma may reflect that passive stretching can be also exerted on other inner ocular tissues within the staphyloma, such as the Bruch membrane, RPE, and retina. This is supported by previous findings that Bruch membrane defect or macular retinoschisis is mostly confined to the area within the staphyloma^27,28^.

Interestingly, when comparing the two most common types of posterior staphyloma, the wide macular and narrow macular type, they did not differ in the CT and ST at any measurement point or curvature index. Based on this, it may be assumed that the narrow macular type is not a less protruded form but an independent type of staphyloma. If the wide macular type is a more protruded form of staphyloma that progresses from the narrow macular type, one would expect further thinning of the choroid and sclera. Each highly myopic eye might have different a location and extent of the region with reduced scleral resistance resulting from altered collagen fibril arrangement^29^, and subsequent scleral outpouching may be confined only within this region, by which the type of posterior staphyloma is classified^8^. However, long-term changes in the type of staphyloma are still unknown, and it should be noted that patients with narrow macular type were significantly younger than those with wide macular type in this study. Although statistical analysis was not performed due to the small number of patients, eyes with combined staphylomas of peripapillary plus narrow macular type in this study had a thinner choroid and sclera compared to the simple wide macular type. This suggests that compound staphyloma might represent a more advanced form of staphyloma. Further longitudinal studies are required to verify the morphological changes in a posterior staphyloma.

This study has some limitations. First, because of the cross-sectional design of this study, it was difficult to determine the causal or temporal relationship between choroidal and scleral thinning in highly myopic eyes. Second, the presence of posterior staphyloma was mainly determined based on ultra-widefield retinal imaging, but this method has been reported to have high sensitivity and specificity for the detection of staphyloma^8^. Although widefield swept-source OCT images were also used to assess the presence of posterior staphyloma, the edge of the deep staphyloma may not be seen due to the limited scan depth. Third, thickness measurement of the extremely thin choroid may be less reliable due to the limited resolution of the OCT, although images were maximally magnified for CT measurement. Therefore, interobserver intraclass correlation coefficients for CT at measurement points with a thinner choroid, such as the subfoveal and nasal regions, were lower than those at other points, but were greater than 0.8, indicating an excellent agreement. Intraobserver agreement was not evaluated, but interobserver agreements for all OCT measurements were excellent in this study.

In conclusion, this study demonstrated that CT and ST are more decreased in eyes with more structural changes of high myopia, especially in the presence of posterior staphyloma. Although the primary tissue of structural change in highly myopic eyes is not fully elucidated, a greater decrease in ST in eyes with posterior staphyloma suggests altered biomechanical properties of the sclera within the staphyloma. Further longitudinal studies will improve our understanding of the mechanism of myopic macular complications from the standpoint of structural change.

## Supplementary Information

Supplementary Information

## Data Availability

The datasets generated during and/or analysed during the current study are available from the corresponding author on reasonable request.
