# Supplementary material for: Decreased choroidal and scleral thicknesses in highly myopic eyes with posterior staphyloma
Source: Sci Rep. 2021 Apr 12;11:7987. doi: 10.1038/s41598-021-87065-6 (PMC8041899; doi:10.1038/s41598-021-87065-6)

# Title: Decreased Choroidal and Scleral thicknesses in highly myopic eyes with posterior staphyloma

Un Chul Park, MD, PhD[1,2], Eun Kyoung Lee, MD[1,2], Bo Hee Kim, MD[1,2], Baek-Lok Oh, MD[1,2]

[1]Department of Ophthalmology, Seoul National University College of Medicine, Seoul, Korea

[2]Retinal Degeneration Research Laboratory, Seoul National University Hospital Biomedical Research Institute, Seoul, Korea

**Corresponding author: Un Chul Park, M.D., Ph.D.**

**Department of Ophthalmology, Seoul National University College of Medicine, 103 Daehak-ro, Jongno-gu, Seoul 110-799, Korea**

**Tel: (+82) 2-2072-2438; Fax: (+82) 2-741-3187; e-mail: ucpark@snu.ac.kr**

**This file contains two supplementary tables and one supplementary figure.**

## Supplementary Table S1. Intraclass correlation coefficients for interobserver variability

| Parameters | | ICC | 95% Confidence Interval | |
|---|---|---|---|---|
| | | | Lower Limit | Upper Limit |
| Choroidal thickness | Subfoveal | 0.924 | 0.901 | 0.941 |
| | Superior | 0.965 | 0.954 | 0.973 |
| | Inferior | 0.950 | 0.932 | 0.963 |
| | Temporal | 0.961 | 0.947 | 0.971 |
| | Nasal | 0.892 | 0.857 | 0.918 |
| Scleral thickness | Subfoveal* | 0.936 | 0.916 | 0.951 |
| | Superior | 0.958 | 0.940 | 0.971 |
| | Inferior | 0.930 | 0.906 | 0.948 |
| | Temporal | 0.958 | 0.940 | 0.971 |
| | Nasal | 0.901 | 0.870 | 0.924 |
| Curvature index (range)* | | 0.920 | 0.896 | 0.938 |

ICC= Intraclass correlation coefficient

*: Excluded eyes with dome-shaped macula

**Supplementary Table S2. Comparison of choroidal and scleral thicknesses between eyes with wide macular and narrow macular type of posterior staphyloma**

| Parameters | Type of posterior staphyloma | | *P*-value |
|---|---|---|---|
| | **Wide macular** | **Narrow macular** | |
| Number of eyes (%) | 102 (60.4%) | 53 (31.4%) | |
| Mean age, years (range) | 65.2 ± 10.0 (38 ~ 85) | 58.8 ± 11.3 (24 ~ 84) | < 0.001 |
| Gender, number of eyes (%) of women | 90 (88.2%) | 42 (79.2%) | 0.135 |
| Mean axial length, mm (range) | 30.5 ± 2.6 (25.7 ~ 37.1) | 31.4 ± 1.7 (26.2 ~ 34.6) | 0.072 |
| Mean baseline BCVA, logMAR (range, Snellen equivalent) | 0.53 ± 0.49 (-0.08 ~ 2.50, 20/68) | 0.59 ± 0.57 (0.00 ~ 2.00, 20/78) | 0.476 |
| Dome-shaped macula | 19 (18.6%) | 11 (20.8%) | 0.750 |
| Choroidal thickness, μm (range) | | | |
| Subfoveal | 29.9 ± 22.9 (0 ~ 133) | 27.0 ± 15.8 (0 ~ 86) | 0.423 |
| Superior | 46.9 ± 34.0 (0 ~ 172) | 47.6 ± 25.2 (0 ~ 140) | 0.902 |
| Inferior | 39.8 ± 31.9 (0 ~ 163) | 37.4 ± 24.9 (0 ~ 110) | 0.653 |
| Temporal | 47.3 ± 43.5 (0 ~ 215) | 36.9 ± 26.0 (0 ~ 142) | 0.117 |
| Nasal | 25.2 ± 19.0 (0 ~ 82) | 23.9 ± 19.5 (0 ~ 88) | 0.797 |
| Scleral thickness, μm (range) | | | |
| Subfoveal* | 279.4 ± 96.4 (108 ~ 542) | 245.8 ± 92.8 (108 ~ 506) | 0.109 |
| Superior | 243.4 ± 79.3 (93 ~ 444) | 216.4 ± 67.9 (95 ~ 402) | 0.105 |
| Inferior | 237.1 ± 65.8 (101 ~ 395) | 218.5 ± 64.0 (105 ~ 469) | 0.093 |
| Temporal | 236.3 ± 84.0 (70 ~ 456) | 208.8 ± 66.7 (100 ~ 469) | 0.060 |
| Nasal | 268.0 ± 71.2 (129 ~ 492) | 250.1 ± 81.2 (83 ~ 477) | 0.119 |
| Curvature index (range)* | 1.047 ± 0.032 (1.008 ~ 1.241) | 1.055 ± 0.032 (1.019 ~ 1.143) | 0.182 |

BCVA = Best-corrected visual acuity; logMAR = logarithm of minimal angle of resolution

*: Excluded eyes with dome-shaped macula

**Supplementary Figure S1. A representative OCT image to illustrate the curvature index. Using ImageJ software (Version 1.47, National Institute of Health, Bethesda, MD; available at https://imagej.nih.gov/ij/), after converting OCT images to a 1:1 µm mode, the length of retinal pigment epithelium line (yellow line) between two points at a 3,000 µm distance from the fovea (6,000 µm apart) on a B-scan image was divided by a straight distance between the two points (red line) to yield a curvature index. In this eye, horizontal curvature index was 1.143.**

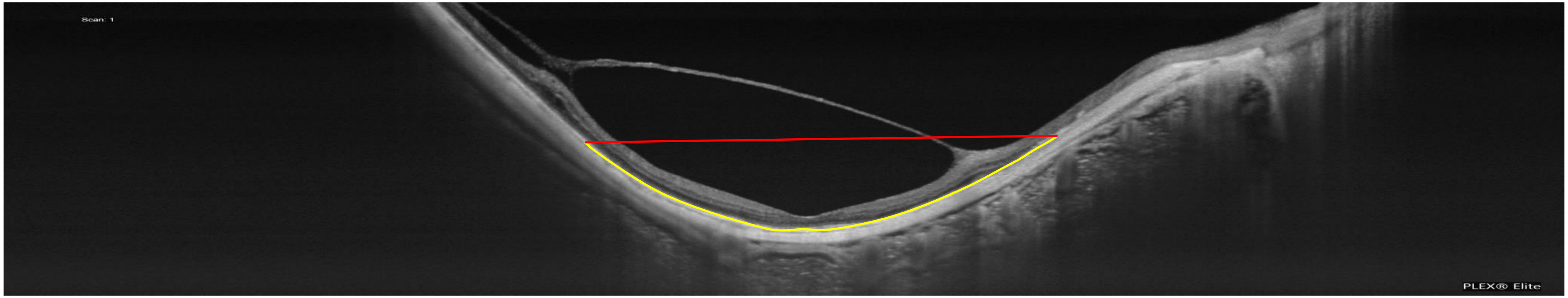

Supplement: Supplementary file 1 — Supplementary Information [file 41598_2021_87065_MOESM1_ESM.pdf]
